# Triadin/Junctin Double Null Mouse Reveals a Differential Role for Triadin and Junctin in Anchoring CASQ to the jSR and Regulating Ca^2+^ Homeostasis

**DOI:** 10.1371/journal.pone.0039962

**Published:** 2012-07-02

**Authors:** Simona Boncompagni, Monique Thomas, Jose R. Lopez, Paul D. Allen, Qunying Yuan, Evangelia G. Kranias, Clara Franzini-Armstrong, Claudio F. Perez

**Affiliations:** 1 DNI-Department of Neuroscience and Imaging, CeSI-Center for Research on Ageing, University of G. D'Annunzio, Chieti, Italy; 2 Department of Cell and Developmental Biology, University of Pennsylvania, Philadelphia, Pennsylvania, United States of America; 3 Molecular Biology Division, Biomedical Research Foundation, Academy of Athens, Athens, Greece; 4 Department of Pharmacology and Cell Biophysics, College of Medicine, University of Cincinnati, Cincinnati, Ohio, United States of America; 5 Department of Anesthesia, Perioperative and Pain Medicine, Brigham and Women's Hospital, Boston, Massachusetts, United States of America; University of Queensland, Australia

## Abstract

Triadin (Tdn) and Junctin (Jct) are structurally related transmembrane proteins thought to be key mediators of structural and functional interactions between calsequestrin (CASQ) and ryanodine receptor (RyRs) at the junctional sarcoplasmic reticulum (jSR). However, the specific contribution of each protein to the jSR architecture and to excitation-contraction (e-c) coupling has not been fully established. Here, using mouse models lacking either Tdn (Tdn-null), Jct (Jct-null) or both (Tdn/Jct-null), we identify Tdn as the main component of periodically located *anchors* connecting CASQ to the RyR-bearing jSR membrane. Both proteins proved to be important for the structural organization of jSR cisternae and retention of CASQ within them, but with different degrees of impact. Our results also suggest that the presence of CASQ is responsible for the wide lumen of the jSR cisternae. Using Ca^2+^ imaging and Ca^2+^ selective microelectrodes we found that changes in e-c coupling, SR Ca^2+^content and resting [Ca^2+^] in Jct, Tdn and Tdn/Jct-null muscles are directly correlated to the effect of each deletion on CASQ content and its organization within the jSR. These data suggest that in skeletal muscle the disruption of Tdn/CASQ link has a more profound effect on jSR architecture and myoplasmic Ca^2+^ regulation than Jct/CASQ association.

## Introduction

The sarcoplasmic reticulum (SR) of skeletal muscle is a differentiated domain of the endoplasmic reticulum [1] that acts as the intracellular Ca^2+^store. The SR has two clearly delimited domains with distinct function, structure and composition: the free SR (fSR) rich in sarco-endoplasmic reticulum Ca^2+^ ATPase (SERCA1) important for Ca^2+^ re-uptake and the junctional SR (jSR), containing among other proteins the ryanodine receptor Ca^2+^ release channels (RyRs) and the Ca^2+^ binding protein calsequestrin (CASQ). The jSR functionally communicates with invaginations of the surface membrane (the transverse tubules, T-tubule) where RyR1 interacts with several protein components forming functional multi-protein complexes defined as the Calcium Release Unit (CRU).

In adult skeletal muscle CRUs are in the form of triads with two jSR cisternae, also called lateral sacs, facing a central T-tubule. In the junctional face membrane of the jSR, RyR1 interacts with Tdn, Jct and CASQ forming a macromolecular complex thought to regulate RyR1 activity [2], [3], [4], [5], [6]. RyR1s are capable of self assembling into ordered arrays in the absence of all other junctional proteins [7] and have a semi-crystalline arrangement at the junctional face of the SR where their cytoplasmic domains are visible as densities located at periodic intervals of ∼30 nm within the junctional gap between T-tubule and SR membrane [8]. CASQ is a low-affinity Ca^2+^binding protein [9], [10], [11] located in the lumen of the jSR that greatly increases the SR Ca^2+^ storage capacity [12], [13], [14]. CASQ has the property of polymerizing into elongated linear polymers in the presence of cations, including Ca^2+^, at physiological concentrations [15]. Polymerized CASQ1 (in fast twitch fibers) and mixed CASQ1 and 2 (in slow twitch fibers) appear in electron micrographs of skeletal muscle jSR, as random aggregates of narrow linear structures cut at all angles, first described as a “delicate meshwork” in frog fibers [8]. Type-2 CASQ also has the same configuration in cardiac muscle after overexpression [16]. It is expected that monomeric CASQ is not directly visible in the EM due to its small size and because of this structural observations do not allow for studies of the ratio of polymer versus monomer at a given point in time or on possible cycling between the two states during a contraction cycle [17].

Junctin [2] and triadin [3], [4], [18] are two intrinsic membrane proteins that are thought to anchor CASQ to the junctional face membrane of the SR as well as to modulate the RyR1 channel function [19], [20], [21], [22]. Both proteins contain binding site for CASQ as well as for RyR1. Tdn forms disulphide-linked oligomers [3], [23], [24], [25] while Jct remains monomeric and can bind only to the luminal domain of the RyR [19], [21]. Ultrastructural evidence for CASQ connection to jSR membrane comes from the observations of lumen-to-membrane links in the native jSR of toadfish [26], and of the condensing effect on CASQ structure by overexpressed Jct and Tdn in cardiac muscle [16], [22].

In addition to being involved in facilitating the cross-communication between CASQ and RyR1 [14], [19], [21], [27] several lines of evidence have suggested that Tdn is also an important regulator of the myoplasmic Ca^2+^ homeostasis in skeletal muscle [28], [29], [30]. Recently, we have shown that this regulatory role may be mediated by modulation of the FKBP12/RyR1 interaction, and that this interaction plays a key role in e-c coupling [31], [32], [33]. Evidence for a role of Jct in Ca^2+^ homeostasis comes primarily from studies in cardiomyocytes where either overexpression [34], [35], [36] or reduction of Jct expression [37] have been associated with alterations of SR Ca^2+^ release and contractility. Studies in C_2_C_12_ myotubes [38] and reconstituted RyR1/Jct/Tdn complexes in bilayer lipid membranes systems [39] suggested that in skeletal muscle Jct not only plays a similar regulatory role as Tdn but it may be more critical than Tdn in maintaining SR Ca^2+^ store size and mediating the signaling between CASQ1 and RyR1.

Taking advantage of the currently existing Jct-null (Jct^−/−^, [37]) and Tdn-null (Tdn^−/−^, [30]) mice we developed a double-null Tdn/Jct mouse to examine the contributions of each of these proteins to the general architecture of the junctional face membrane and their role, separately and in combination, on EC coupling and SR function. The structural study indicates a major role of Tdn in providing periodic *anchoring* of the CASQ polymer to the jSR membrane and a synergistic effect of both Tdn and Jct (but with a predominance of Tdn) in stabilizing the CASQ polymer within the jSR vesicles. Consistent with their corresponding effects on jSR CASQ retention Tdn-null cells showed a reduced e-c coupling efficiency, but the lack of Jct had very limited, if any, functional effects.

## Results

### Protein expression profiles

#### Jct-null and Tdn-null muscles display dissimilar protein expression profile of key CRU components

**Figure 1:**
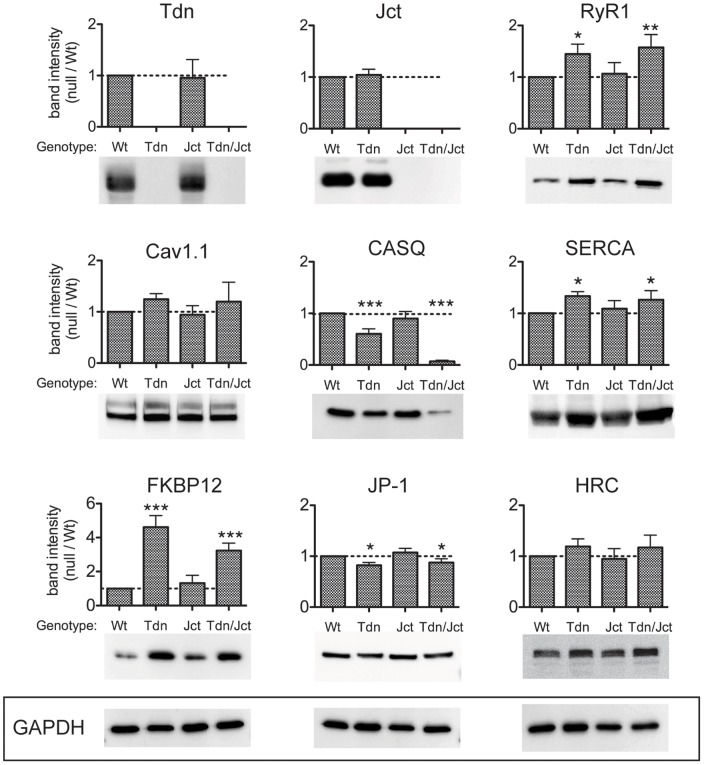
Relative levels of CRU proteins in crude homogenates from skeletal muscle. Identical amounts (25 µg/lane) of microsomal fraction of skeletal muscles from WT, Tdn-null, Jct-null and Tdn-/Jct null mice were loaded and immunoblotted with several antibodies. Membranes were tested for expression of triadin (Tdn), junctin (Jct), ryanodine receptor (RyR1), Dihydropyridine receptors (Cav1.1), Calsequestrin (CASQ), SERCA-1 pump (SERCA), FK506 binding protein (FKBP12), Junctophilin-1 (JP-1) and Histidin-rich Ca^2+^ binding protein (HRC) as described in Material and Methods. Band intensity for each protein was normalized to GAPDH expression to correct for loading and plotted as fraction of its WT counterpart (dotted line). Data presented as mean ± SD of 3–7 independent blots. *p<0.05, **p<0.01, ***p<0.001 (ANOVA, One-way analysis of variance, Tukey's multiple comparison test). Representative blots for each series, including three anti-GAPDH blots (lower panel), are presented.

To assess the effect of the absence of Jct, Tdn and Tdn/Jct on the relative expression levels of several CRU components we examined crude membrane preparations of WT, Tdn-null, Jct-null and Tdn/Jct double-null from hind leg muscles using Western blots analysis. Jct-null and Jct-null muscles did not significantly differ from WT in relative expression levels of any CRU proteins examined (RyR1, Ca_V_1.1, Tdn, FKBP12, SERCA-1, CASQ, Junctophilin-1 [JP-1] and Histidine-rich Ca^2+^ binding protein, HRC) (Fig. 1). Tdn-null muscles, on the other hand, displayed statistically significant up-regulation of RyR1 (∼45%), SERCA-1 (∼30%) and FKBP12 (∼460%) and a significant down-regulation of CASQ (∼40%) and JP-1 (∼18%) (Fig. 1), consistent with previous data [30]. Although Ca_V_1.1 and HRC expression levels appeared slightly elevated in Tdn skeletal muscles the increase was not statistically significant. Importantly, unlike the case in the heart [40] it appears that the absence of Tdn did not affect Jct expression levels in skeletal muscle.

The expression profile of CRU proteins of Tdn/Jct-null muscles closely resembles that of the Tdn-null muscle showing a similar up-regulation of RyR1 (∼55%), SERCA-1 (∼28%) and FKBP12 (∼320%) and down-regulation of JP-1 (∼10%) (Fig. 1). Interestingly, the reduction in CASQ expression in the double null muscles is considerably more dramatic than in Tdn-null muscles, reducing levels to only 7% of WT. As in Tdn-null muscle, there appeared to be a small, but statistically insignificant increase in Ca_V_1.1 and HRC expression in double null muscles. Altogether, these results suggest that the absence of Jct alone had a minimal effect on the expression profile of its other CRU partners. However, in the absence of Tdn the lack of Jct had a dramatic effect on expression of CASQ.

### Structural alterations

#### The fine structure of jSR cisternae in skeletal muscle fibers from WT mice

In order to relate the structural changes to specific fiber types, we used three types of muscles. Mouse EDL contains a majority of fast twitch type IIB fibers [41], [42]; the sternomastoid a majority of fast twitch type IIX fibers [43]. These two muscles are composed of fast twitch fibers that not only share equivalent structure but also displayed similar type and levels of structural alterations in all mutated mice. Because of this, for documentation purposes, both types of muscle were used as examples of fast twitch fibers. The soleus contains mostly slow twitch fibers and fast twitch type IIA [41].

**Figure 2:**
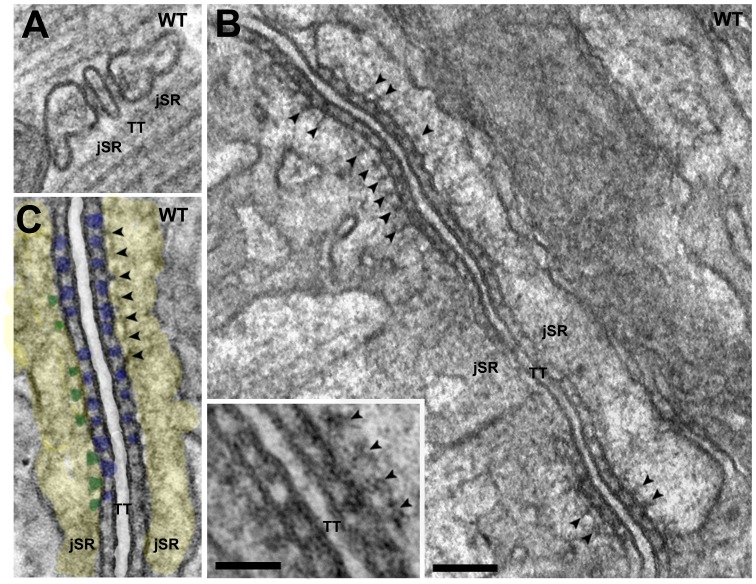
Images from thin sections at right angle to the T-tubule long axis (A) and parallel to it (B, C and inset) from WT EDL muscles. A) In WT muscles the two jSR cisternae (jSR) facing the central T-tubule (TT, white space) are relatively large, they contain electron dense polymer of CASQ and are joined to T-tubules by two feet. B, C and inset). A row of periodically disposed feet (RyRs, blue dots in C) fills the jSR-T tubule junctional gap and the profiles of small *anchors* (green in C) project into the jSR lumen (yellow in C) in a position alternate to that of feet (arrowheads in C, better seen in the inset). The distal ends of *anchor* connect to a thin linear density, particularly prominent in C, presumably a long CASQ polymer. *Bars: A–C, 0.1 *µ*m; inset, 0.05 *µ*m.*

**Figure 3:**
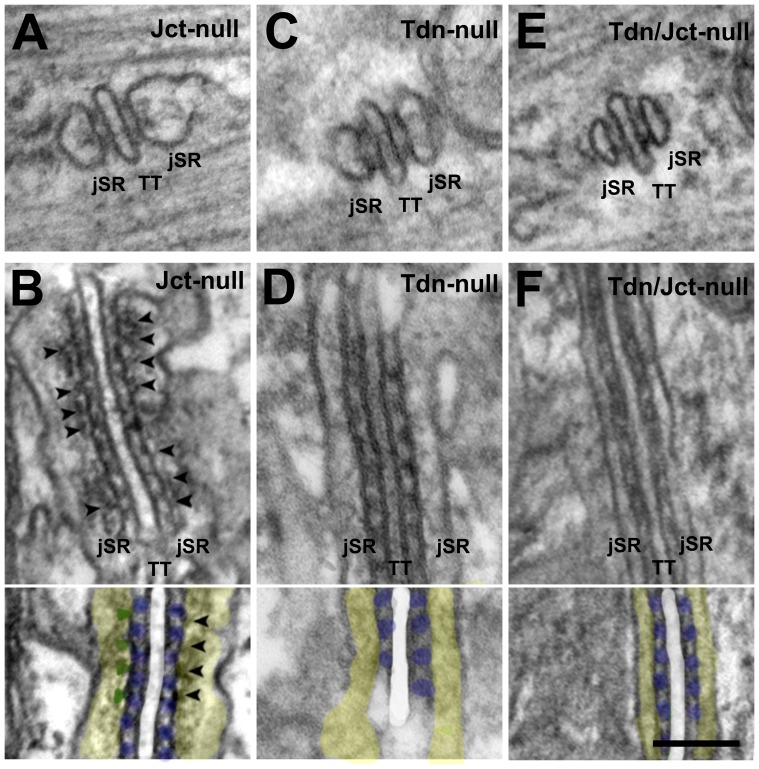
Sections from sternomastoid muscle in mutated mice. A and B) In the absence of Jct the overall structure is not visibly altered. A polymer of CASQ fills the jSR and is *anchored* to the feet-bearing jSR membrane (arrowheads). The size of the transversely cut jSR profiles is slightly smaller than wild type in this image (see detail in Fig. 4C and D). In the absence of Tdn *anchors* are missing and the visible jSR content quite reduced although still visible. The jSR cisternae are considerably smaller. E and F) In the double mutant, the jSR profiles are very narrow and they seem to be basically empty. In all cases, the jSR-T tubule junctional gap and the rows of feet are unaltered. *Colors:* white: T-Tubule; yellow: jSR lumen; blue RyRs; Green: anchors. *Bar: A–F, 0.1 *µ*m.*

The ultrastructure of a skeletal muscle triad consists of a central T-tubule profile (TT) flanked by two jSR cisternae that are joined to it by two SR feet (RyRs) on either side (Fig. 2 A). In a section that cuts along the long axis of the triad, the feet are located at center to center distances of ∼30 nm along even rows (Fig. 2 B, C and inset). CASQ is located in the jSR lumen in proximity of the feet [5], [6], and in thin sections for electron microscopy it appears as a complex matrix (Fig. 2) whose structure is consistent with that expected from thin sections through a three-dimensional network of randomly disposed long, thin polymers [15]. In the SR, CASQ polymer constitutes the electron dense background detectable in the cisternae, visualized as a fine meshwork filling the entire jSR cisternae (Figs. 2 and 3, yellow).

A structural detail that has been poorly emphasized in the past is the presence of noticeable periodically disposed electron densities, *anchors*, (Fig. 2 C and B, arrowheads) directly connecting the CASQ filaments to the jSR membrane. Importantly the *anchors* are located at distances matching those between the feet and join the membrane exactly at the electron translucent space between the latter. An additional detail is a fine line parallel to the jSR membrane that appears to connect the luminal ends of the *anchors* to each other and to the rest of the CASQ network, best visible in Fig. 2 C. The length of the *anchors* measured from the edge of the lipid SR membrane to the fine line is 4.3±0.7 nm (n = 29 measurements, 4 mice). The line is of the same general appearance as those constituting the randomly disposed linear CASQ polymers. Periodic *anchors* and *lines* were observed in all muscles from WT mouse analyzed in this study.

#### Structural changes resulting from lack of Jct and Tdn

Since the expression level of either Tdn or Jct is not affected by the absence of the other, the single and double null mutants mice offered the unique opportunity of clearly distinguishing the specific structural functions of the two proteins. The loss of Tdn, Jct and both proteins did not affect either the overall appearance of the junctional gap between the membranes of SR and T-tubules or the frequency and disposition of feet within it, but differentially and sometimes profoundly affected the architecture of the jSR lumen.

With the absence of Jct the internal structure of jSR cisternae does not appear obviously altered: the periodic *anchors*, the fine line connecting the *anchors* to the CASQ network and the network itself are still present (Fig. 3 A and B).

In Tdn-null muscles, on the other hand, both the structure of the luminal content and the volume of the jSR cisternae are significantly altered (Fig. 3 C and D). In addition although the electron dense gel matrix of CASQ is still somewhat visible and slightly structured, this structure is mostly quite weak and not well defined (Fig. 3 D). Most noticeable is the fact that the periodically disposed *anchors* and the fine line connecting them, usually present in close proximity to the junctional face membrane, are not detectable at all. This effect was consistent in all muscles analyzed.

The effect of double deletion (Tdn/Jct-null) on jSR structure is more profound than that of deletion of Tdn alone. In this case jSR profiles show no evidence of any internal substructure, although they show a slight diffuse density, and they are quite narrow (Fig. 3 E and F and below).

**Figure 4:**
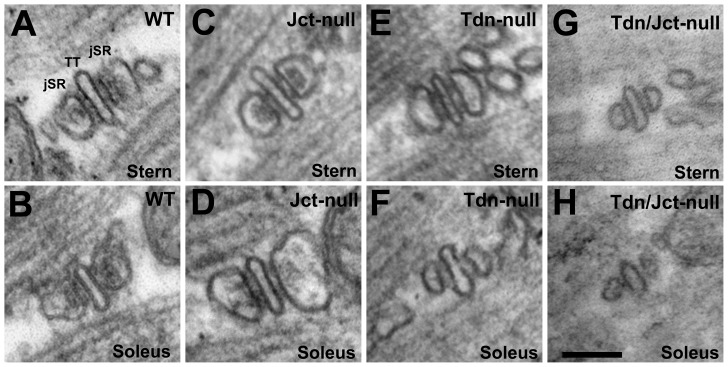
Sections at right angle to triads in sternomastoid (top row) and soleus (bottom row) muscles illustrating changes in dimensions of the jSR cisternae relative to WT. Compare with Table 1. C and D) In Jct-null muscles the triads are slightly smaller than WT in sternomastoid (A), but somewhat larger in soleus (compare B and D; E and F) in Tdn-null fibers the jSR cisternae are smaller in all muscles; G and H) in the double null the dimensions are further reduced. *Bar: A–H, 0.1 *µ*m.*

In parallel to the structural changes, there are noticeable alterations in jSR volume. The area occupied by the jSR profiles in sections cut at right angles to the triad long axis is directly proportional to the jSR volume. In Jct-null muscles the jSR area is decreased by ∼27% relative to WT (Fig. 4 A and B) in sternomastoid (Fig. 4 C), but increased by ∼18% in soleus (Fig. 4 D). Changes in both muscles are statistically significant (Table 1). In Tdn-null muscles the change is more substantial and the jSR volume is significantly reduced in both muscles (by ∼55% in sternomastoid, Fig. 4 E and by ∼70% in soleus, Fig. 4 F and Table 1). Finally, in the double nulls the average decrease in volume is even larger than in Tdn-nulls, (∼78% for sternomastoid and 81% for soleus, Fig, 4 D and H and Table 1). The sample variance is fairly large in WT and Jct-null muscles, but it is considerably less in Tdn-null and double null fibers from both sternomastoid and soleus, indicating that the cisternae are uniformly small in these samples.

**Table 1:** jSR areas measured in sections at right angle to the T-tubule long axis.

Genotype	Muscle type	No. of junctions (No. of mice)	&jSR cross-sectional area (nm^2^)
**WT**	Sternomastoid	172 (3)	5087±1288
**Jct-null**	Sternomastoid	188 (3)	3658±1166
**Tdn-null**	Sternomastoid	198 (4)	2310±836
**Tdn/Jct-null**	Sternomastoid	149 (3)	1640±589
**WT**	Soleus	115 (2)	5901±2191
**Jct-null**	Soleus	269 (2)	6934±2400
**Tdn-null**	Soleus	98 (2)	1198±495
**Tdn/Jct-null**	Soleus	198 (2)	1127±370

& *mean ± SD.*

*Student's t test: sternomastoid muscles, differences between all categories are very highly significant (P<0.0001). Soleus muscles, differences between WT to Jct-null and Tdn-null to Tdn/Jct-null (P = 0.001 and 0.06 respectively); WT versus Tdn-null; WT versus Tdn/Jct-null; Jct-null versus Tdn-null and Jct-null versus Tdn/Jcn-null (P<0.0001).*

**Figure 5:**
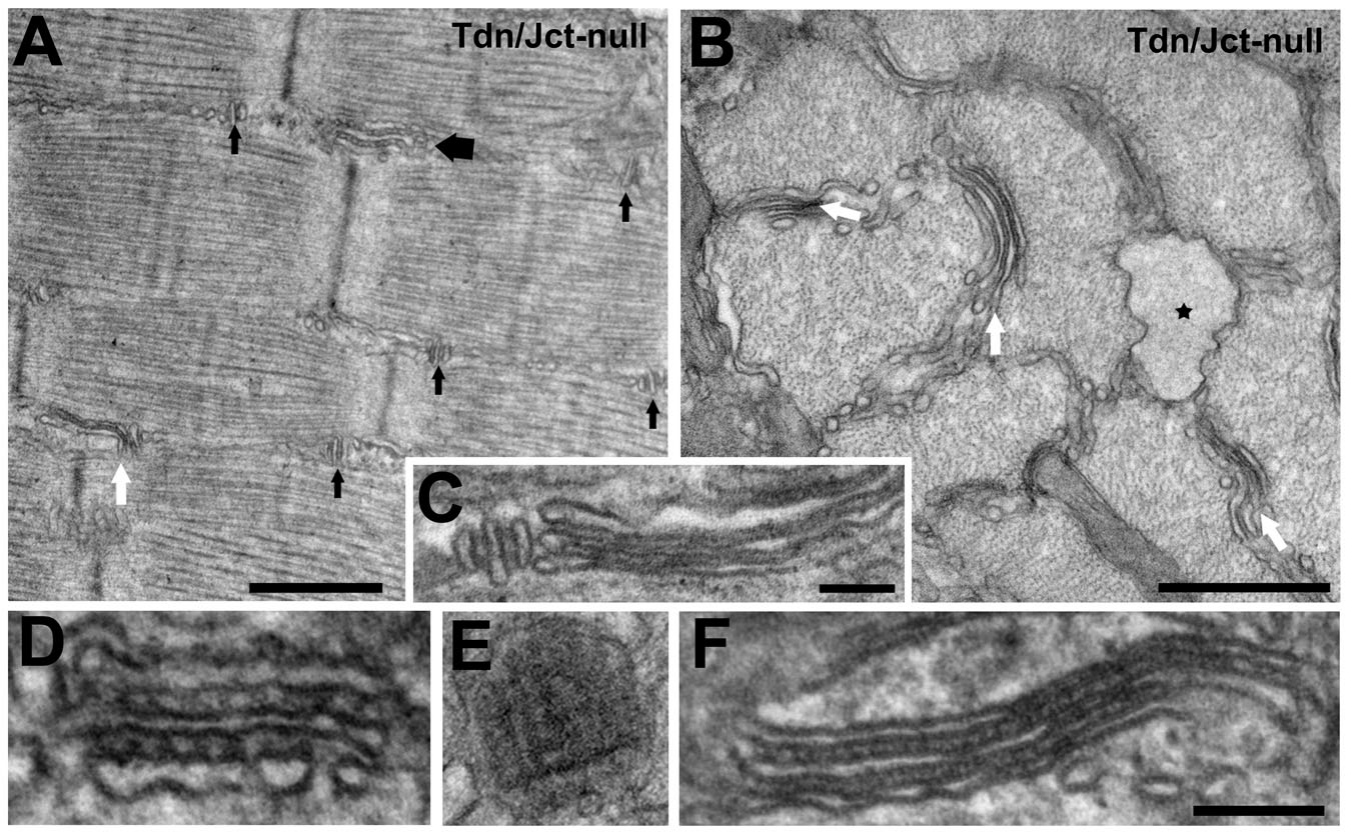
Additional structural alterations: a shift of triads orientation from transverse to longitudinal in fast twitch fibers of EDL and sternomastoid (A, larger arrow) resulting in the development of large jSR plaques carrying multiple rows of feet (D and E). This occurs in fast twitch fibers of Tdn-null fibers, as previously reported, and in the double null mutants. B) The double null mutant fibers show a small number of quite large sacs always located in correspondence of the Z-line (not shown) and filled with a finely granular material similar to the CASQ content of the jSR (star) and some flat SR cisternae (A, B, white arrows, C and detail in F). The flat SR cisternae are separated by small densities that are clearly different from feet (compare D and F, at the same magnification). *Bars: A, 0.5 *µ*m; B, 0.5 *µ*m; C, 0.1 *µ*m; D–F, 0.1 µm.*

An additional alteration of the SR found only in fast fibers of the double null muscles, is the presence of large cisternae at the level of the Z line filled with a content that is identical to that of the jSR cisternae and thus probably represents polymerized CASQ (Fig. 5 B, star). These cisternae are present in approximately 35–40% of fiber profiles seen in cross sections.

#### Additional structural alterations

As previously reported, [30], [44], the orientation of triads in Tdn-null muscles is frequently longitudinal rather than transverse. The effect is also present in the double null muscles (Fig. 5 A larger arrow) and it occurs only in fibers from sternomastoid and EDL, but not from soleus. The inference is that type IIB/IIX fibers are predominantly affected, but type IA and IIA are not. The jSR surface in the shifted triads is circular or oval rather than elongated and for that reason feet are gathered into wide plaques, while maintaining their normal spacing (Fig. 5 E). The direct result of this shape change is that each jSR plaque contains a larger number of RyRs in the longitudinally oriented than in the transverse triads (Fig. 5 D and E), thus accounting for an increased expression level of RyRs in Tdn-null and double null muscles (see Fig. 1). The changing in position from transversal to longitudinal of jSR cisternae and triads is a common reaction of fast twitch fibers to a variety of pathological stimuli like brief or prolonged denervation [45], [46] and the lack of CASQ [47]. This is in contrast to the effect of the absence of Tdn on myocardium that results in a reduction of RyR2 and SR-T tubule junctions [40]. Conversely, Jct does not affect either the position of triads or the expression levels of RyR1 in skeletal muscle, confirming its less dominant role in defining the jSR architecture.

In parallel to the shift in triad position, fibers from Tdn-null and Tdn/Jct double null muscles have an unusual accumulation of flat SR cisternae with an empty lumen (Fig. 5 A, white arrow, B, C and F). These cisternae are continuous with the remaining SR, but not with T-tubules and they are specifically present only opposite the I-Z-I level of the sarcomere indicating that they are derived exclusively from the I band SR. Small electron dense bridges connect the parallel surfaces of adjacent cisternae (Fig. 5 F). These densities are not “feet” (RyR1) profiles for two reasons: first the spacing between them (6.1±0.9 nm, n = 24 measurements, 2 mice) is much closer than the one between RyRs (27±4 nm, n = 72 measurements, 2 mice). Secondly, the distance between the apposed SR and T-tubule membranes in the triad, measured from the centers of the bilayers is 18±2 nm (n = 84 measurements, 2 mice), while the distance between the apposed membranes of the flat SR cisternae is 13±2 nm (n = 51 measurements, 2 mice). Because the flat cisternae are present only in EDL but not in sternomastoid and soleus, it suggests that only type IIB fibers may be involved and are similar to the regularly arranged SR-SR bridges in the tubular aggregates of aging mouse muscle [48] and in denervated muscle [49].

### Functional alterations

To correlate the extent of the jSR alterations of each phenotype with its corresponding effect on Ca^2+^ homeostasis and e-c coupling we conducted Ca^2+^ imaging studies on cultured myotubes from all four genotypes. Cells were analyzed to compare their ability to support both depolarization-induced (e-c coupling) and caffeine-induced Ca^2+^ release, as well as their ability to modulate total SR Ca^2+^ content and myoplasmic resting free Ca^2+^ concentration. In spite of obvious structural and functional differences between cultured myotubes and adult fibers, myotubes were chosen for the current work based on our previous studies in the Tdn-null model. Those studies showed that the behavior of cultured myotubes closely resembled the behavior of adult muscle fibers in terms of e-c coupling efficiency, caffeine-induced Ca^2+^ release, SR Ca^2+^ content and cytoplasmic resting calcium concentrations [30]. Therefore, because of the convenience of being able to perform testing in non-contracting cells, alleviating the possible effects on Ca^2+^ transients as a result of using BTS to prevent contraction, we chose myotubes and not adult muscles to make physiologic measurements in the current study.

#### Depolarization-induced Ca^2+^ transients

**Figure 6:**
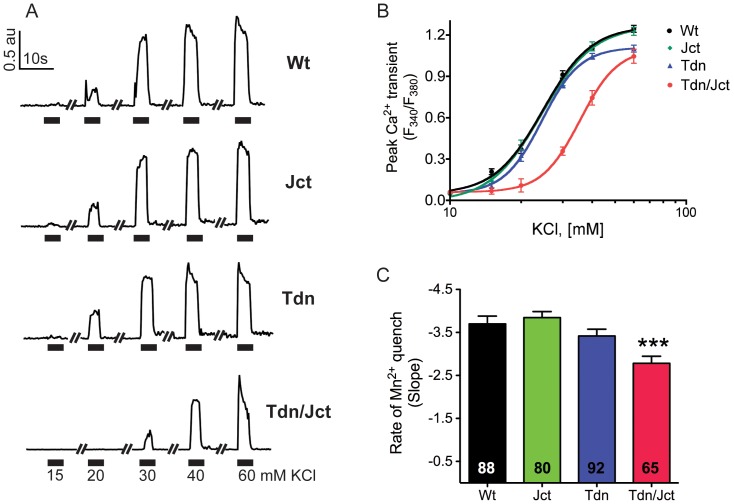
Effect of Tdn and Jct on e-c coupling and ECCE. A) Representative traces of K^+^ -dose responses of primary cultured myotubes from wild type (WT), triadin-null (Tdn), junctin-null (Jct) and triadin/juctin double-null (Tdn/Jct) mouse muscles. B) Average peak fluorescent amplitude of depolarization-induced Ca^2+^ release response of WT (black, n = 114 cells), Jct-null (green, n = 83 cells), Tdn-null (blue, n = 57 cells) and Tdn-/Jct null (red, n = 68 cells) myotubes. Myotubes were loaded with 5 µM Fura-4F and exposed to increased concentrations of K^+^ for 5 s. C) Average rate of decrease in Fura-2 signal by Mn^2+^ quench during depolarization with 80 mM KCl. Numbers in the bars indicate the number of cells analyzed per condition. The data are from 3–4 experiments and are presented as mean ± SEM (***p<0.001 (ANOVA, One-way analysis of variance, Tukey's multiple comparison test)).

In response to exposure to stepwise increases in KCl, Jct-null myotubes showed a classic sigmoidal dose response which was undistinguishable from WT cells both in the peak amplitude of Ca^2+^ transients and the sensitivity to K^+^ (Fig. 6 B). As previously reported [30], [32], Tdn-null myotubes displayed a slightly but significantly smaller peak Ca^2+^ amplitude than WT cells (peak 340/380 ratios of 1.10±0.03 n = 57 cells and 1.24±0.003, n = 114 respectively, mean±SEM, p<0.05) with no evident change in K^+^ sensitivity (Fig. 6 A and B). Myotubes from Tdn/Jct double-null mice showed a reduction in peak amplitude (1.04±0.05 n = 68) comparable to that observed in Tdn-null cells and were significantly less sensitive to K^+^ depolarization as indicated by the rightward shift in K^+^ EC_50_. (Fig. 6 A and B, p<0.001).

#### Excitation-Coupled Ca^2+^ entry (ECCE)

To evaluate the potential role of extracellular Ca^2+^ entry to the global Ca^2+^ signal induced by depolarization we measured ECCE in all groups of cultured myotubes using Mn^2+^ quench studies, as a surrogate measure of Ca^2+^ entry. As shown in Fig. 6 C, the average rate of Mn^2+^ quench in Jct-null and Tdn-null myotubes was not significantly different than in WT cells (p>0.05). Tdn/Jct double-null cells, on the other hand, displayed a small but statistically significant reduction (p<0.01) in the rate of Mn^2+^ quench when compared to WT myotubes. These results suggest that structural alterations of the jSR induced by lack of Tdn and Jct expression had only a minor effect on ECCE. The fact that peak amplitudes of K^+^-induced Ca^2+^ transients of Tdn-null and Tdn/Jct-null myotubes are not statistically different strongly suggests that the reduction in ECCE observed in Tdn/Jct-null cells had a negligible effect on the global Ca^2+^ signal induced by depolarization.

#### Caffeine-induced Ca^2+^ transients

**Figure 7:**
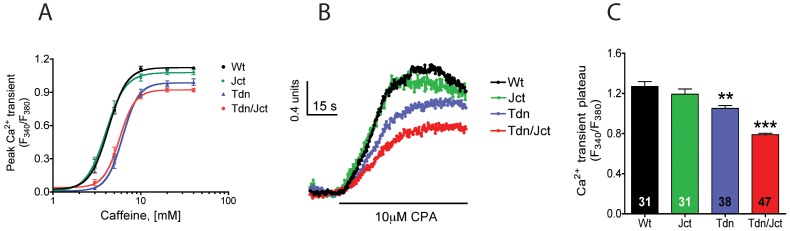
Effect of Tdn and Jct ablation on SR Ca^2+^ content of cultured myotubes. A) Average peak fluorescent amplitude of caffeine-induced Ca^2+^ transients of Fura-4F loaded myotubes from WT (black, n = 59 cells), Tdn-null (blue, n = 52 cells), Jct-null (green, n = 60 cells) and Tdn-/Jct null (red, n = 59 cells) mice. B) Representative traces of CPA-induced Ca^2+^ transients of WT (black), Jct-null (green), Tdn-null (blue) and Tdn/Jct-null (red) myotubes loaded with Fura-2 used to estimate SR Ca^2+^ content. C) Comparison of average peak Ca^2+^ transient amplitude induced by 10 µM CPA. Numbers in the bars indicate the number of cells analyzed per condition. Data presented as mean ± SEM. **p<0.01, ***p<0.001 (ANOVA, One-way analysis of variance, Tukey's multiple comparison test).

To assess the direct effect of absence of either protein on RyR1-mediated Ca^2+^ release we compared the caffeine-induced Ca^2+^ release in Fura-4F loaded myotubes from each phenotype. Jct-null cells displayed average caffeine dose responses curves that closely resembled that of WT myotubes (Fig. 7 A), with peak 340/380 ratio amplitudes at 40 mM caffeine of 1.12±0.01 (n = 59 cells) for WT and 1.08±0.02 (n = 60 cells) for Jct-null cells (p>0.05) and similar EC50: EC50_WT_ 5.0±0.3 mM vs EC50_Jct_ 4.7±0.3 mM (p>0.05). By comparison, Tdn-null cells showed both a significant reduction in peak Ca^2+^ release amplitude (0.99±0.03 [n = 52 cells], p<0.05 compared to WT) and a noticeable rightward shift in caffeine sensitivity (EC50_WT_ 5.0±0.3 mM vs EC50_Tdn_ 6.5±0.4 mM, p<0.01). Tdn/Jct double-null myotubes displayed an even greater reduction in peak caffeine induced Ca^2+^ transient amplitude (0.92±0.02, n = 59 cells p<0.05 vs WT and Tdn-null myotubes). Caffeine EC50 was shifted to the right compared to WT but was unchanged relative to Tdn-null (EC50_Tdn/Jct_: 6.4±0.3 mM, p<0.05).

#### SR Ca^2+^ load

SR Ca^2+^ content of cultured myotubes was estimated from the Ca^2+^ signal obtained by emptying SR stores with the SERCA pump inhibitor cyclopiazonic acid (CPA). Fig. 7 B shows representative Ca^2+^ release traces of Fura-2 loaded myotubes challenged with 10 µM CPA. Average peak 340/380 ratios values for WT, Jct-null, Tdn-null and Tdn/Jct-null myotubes (Fig. 7 C) are; 1.27±0.05 (n = 31 cells), 1.19±0.05 (n = 38 cells), 1.05±0.03 (n = 31 cells) and 0.79±0.01 (n = 47 cells), respectively. By comparison to WT cells these values correspond to reduction of SR Ca^2+^ load of 6% (p>0.05), 17% (p<0.01) and 38% (p<0.001). These data support the hypothesis that there is a significant reduction of SR Ca^2+^content in Tdn-null and Tdn/Jct-null but not Jct-null myotubes, and seems consistent with the caffeine-induced Ca^2+^ release data and the relative CASQ expression levels observed in each phenotype.

#### Myoplasmic resting free Ca^2+^ concentration

Resting Ca^2+^ concentrations for each phenotype were determined in cultured myotubes by direct measurement with Ca^2+^ selective microelectrodes (Table 2). As previously reported [28], [30] under resting conditions primary cultured Tdn-null myotubes had significantly higher [Ca^2+^]_rest_ than WT myotubes (188±12 nM vs 118±4 nM for Tdn-null and WT respectively). By comparison, Jct-null myotubes had a modest, although significant, increase in [Ca^2+^]_rest_ to 136±7 nM while [Ca^2+^]_rest_ in double null myotubes (255±8 nM) was significantly more elevated than Tdn-null (p<0.001).

**Table 2:** Resting cytoplasmic Ca^2+^ levels.

GENOTYPE	[CA^2+^]_REST_ nM (mean ± SD)	*n*	*p*
**WT**	118±4	27	
**Jct-null**	136±7	16	<0.001
**Tdn-null**	188±12	22	<0.001
**Tdn/Jct null**	255±8	22	<0.001

*One-way ANOVA, analysis of variance (nonparametric) with respect to WT cells.*

## Discussion

### Contributions of Tdn and Jct to *anchors*

CASQ polymer within the jSR cisternae is anchored to the RyR-bearing jSR membrane by periodically disposed electron opaque densities (*anchors*). The disappearance of the *anchors* in Tdn-null muscles, while Jct and CASQ are still present, is a direct indication that Tdn is the protein responsible for CASQ anchorage. Identification of Tdn and not CASQ as the major component of the *anchor* is quite consistent with the fact that *anchors* are present in the junctional face membrane of CASQ1-null fast twitch fibers [47]. Nonetheless, the dimming of anchors in Jct-null muscles seems to suggest at least some contribution of this protein to the anchors structure.

**Figure 8:**
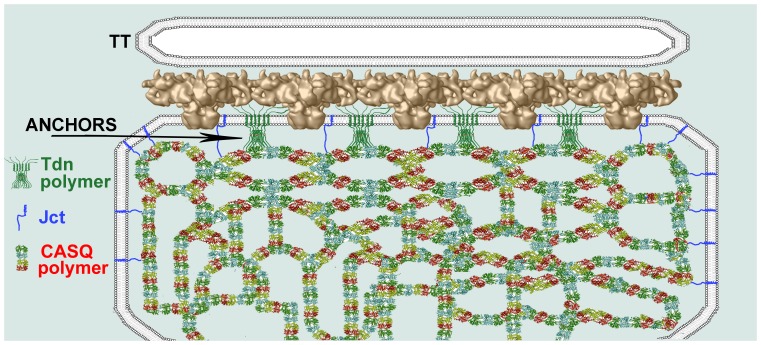
A model proposing the contribution of Tdn's to the jSR anchors and a possible positioning of Jct. In the model the triad is seen in a view parallel to the T-tubule axis (as in Figs. 2 B–C and 3 B, D and F) and the RyR array is modeled as seen in this orientation. The proportion between RyR heights and their spacing is appropriate, as suggested to us by Dr M. Samso [64]. Clusters (polymers) of six Tdn molecules, modeled roughly according to the topology proposed by Knudson *et al*., (1993) and Marty *et al*., (1995) are located between the RyRs as initially suggested by Fan *et al*. (1995). Their aggregated mass is responsible for the visible anchors, but the exact ratio of Tdns to anchors is not known. Individual triadin molecules are connected to RyRs and to a long linear CASQ polymer on the luminal side of the SR. The latter corresponds to the fine line visible in the EM images at the tips of anchors (see Fig. 2 C and inset). The jSR lumen is filled by long CASQ polymers that randomly intersect each other [15]. Junctin is depicted as monomers associated with RyRs [19], [21]. Although we cannot visualize them directly, it is likely that individual Jct molecules are positioned as indicated along the jSR face and also possibly at the sites where CASQ is linked to the lateral sides of the SR.

Two relevant questions are whether Tdn alone can fully account for the visible *anchors* and whether their periodic positioning is consistent with known Tdn/RyR interactions [3], [19]. In answer to the first question, we note that the average length of *anchors* (∼4 nm) is consistent with two alternate models of the protein [4], [50]. We expect that the cytoplasmic extensions of individual triadins are too small to be visible. However, Tdn is bundled into higher order structures by S-S bonds, [4], [23], [50], [51] and clusters of triadin molecule tails of the type depicted in Fig. 6 of Fan *et al*., 1995 may constitute the visible *anchors*. This is depicted in the model (Fig. 8) that also shows the presumptive connection of Tdn to extended CASQ polymers parallel to the jSR-membrane.

Regarding the second question, whether RyRs affect the positioning of *anchors*, we notice that *anchors* are present in dyspedic RyR-null fibers, but their disposition is clearly less periodic than in WT fibers [52]. Thus association of Tdn with RyR may not be necessary for the formation of *anchors*, but the periodic positioning of *anchors* is determined by the presence of RyRs.

### CASQ content and jSR volume. Are Jct and Tdn responsible for CASQ retention in the jSR?

Decrease in jSR cisternae size, the loss of visible content and expression levels of CASQ follow the same trend: all are hardly changed in Jct-null; significantly reduced in Tdn-null and greatly reduced in Tdn/Jct-null. This ties CASQ expression levels (an indirect indication of the protein stability) to its retention within the jSR and establishes a correlation between volume of the jSR cisternae, visibility of the CASQ mass in the electron microscope as a structured coil of protein and the content of polymerized CASQ. Extending the observation of monomer to polymer transition in the movement of CASQ from the rough ER to the jSR [53], [54], we suggest that Tdn and Jct provide not only anchoring but also stability to polymers of CASQ. A recent study in C_2_C_12_ myoblasts seems to confirm this idea by showing that Jct plays an important role in depolymerization dynamics of cardiac calsequestrin (CASQ2) upon depletion of Ca^2+^ stores [55]. However, whether the differential effect that Tdn and Jct had on the visible content of CASQ in jSR cisternae seen in the current study is the result of differential effects of each protein on CASQ polymer stability still needs to be directly determined.

In skeletal muscles Tdn is the prevalent CASQ retaining protein and in our view anchors are to be considered initiation sites for its polymerization while Jct has a less critical role, but both on their own can retain a portion of CASQ within the jSR. The very reduced size of jSR cisternae and the presence of large SR sacs containing what appears to be polymerized CASQ in fibers of the double null muscles suggests that CASQ protein may not be retained within the jSR at all when both proteins are missing.

### Role of Tdn and Jct on Ca^2+^ homeostasis

Although cultured myotubes do not exactly replicate adult muscle physiology in our previous studies in Tdn-null mice we showed that the same functional features analyzed in this study (e-c coupling efficiency, SR Ca^2+^ load and [Ca^2+^]_rest_) were equivalent in myotubes and adult muscle fibers [30]. Our data revealed a direct correlation between the severity of the structural perturbations and the extent of functional alterations. Indeed, in all three genotypes the efficiency of e-c coupling and reduction in SR Ca^2+^ load parallels the reduction in CASQ content and jSR volume. Importantly, we show that in Tdn-null the well-defined phenotype is accompanied by a seemingly total loss of CASQ achoring to the jSR. The effects of Tdn ablation on e-c coupling and SR Ca^2+^ content have been associated with hyperactivation of RyR1 as a result of disruption of the FKBP12/RyR1 interaction [28], [30], [32]. These effects were partially but not fully reverted by expression of FKBP12.6, suggesting that additional modulators of RyR1 may be involved in dysregulating RyR1 activity [32]. The loss of CASQ anchoring observed in Tdn-null muscle strongly suggest that a lack of CASQ-mediated regulation of RyR1 may also be involved in dysregulation of Ca^2+^ homeostasis in these cells.

Simple lack of Jct expression did not result in significant alterations of e-c coupling signaling, caffeine-induced Ca^2+^ release or SR Ca^2+^ content, or the expression levels of key jSR proteins, including CASQ. This result is in disagreement with a previous study in C_2_C_12_ cells where acute knockdown of Jct expression, was shown to cause a significant reduction of both SR Ca^2+^ store size and K^+^-induced Ca^2+^ release [38]. Our data instead show that the disruption of the Tdn/CASQ complex has a much greater impact on global myoplasmic Ca^2+^ homeostasis than the disruption of the Jct/CASQ complex. This result also contrasts with studies in lipid bilayer systems reconstituted with purified RyRs followed by the adding back of Jct or Tdn [39] which found that Jct has a predominant role over Tdn on relaying the functional interaction between RyR1 and CASQ1 in skeletal muscle. Based on our previously published bilayer studies using native RyR1 containing SR membranes indicating that lack of Tdn expression has a dramatic effect on RyR1 channel behavior [28] as a result of impaired RyR1/FKBP12 interaction, and the findings of the current study it appears that in intact cells the effects of Tdn expression had a greater impact on resting calcium than that mediated by Jct.

All nulls show elevated [Ca^2+^]_rest_. In the case of Tdn-null and Tdn/Jct double-null cells we attribute this to the consequences of a reduction of SR stores caused by RyR1-mediated SR Ca^2+^ leak [28], [30]. The degree of this elevation correlated well with total SR Ca^2+^ load confirming that hypothesis. The observations in the double null phenotype suggest that although Jct cannot compensate for the lack of Tdn it does contribute to restrict the deleterious effects of the Tdn-null phenotype, supporting a role for Jct in regulating Ca^2+^ homeostasis in skeletal muscle. However, because of the targeting strategy used to knockout junctin may also prevent expression of aspartyl-**β**-hydroxylase (Asph), humbug and junctate [56], [57], [58], [59]. The use of an antibody against homologous region of the N-termini of the three proteins reveled that expression of junctate in Jct-null mouse has not been altered in cardiac muscles however, the N-termini antibody failed to detected expression of Asph/humbug. Whether the enzymatic activity of Asph/humbug has any role in Ca^2+^ cycling regulation of cardiac or skeletal muscles is unknown but because of this nonspecific effects on [Ca^2+^]_rest_ as a result of Jct ablation can not be ruled out.

Overall, our study indicates that in skeletal muscle Tdn plays a more critical role than Jct in defining the structural architecture of the jSR and identifies Tdn as the preferred anchor points for CASQ. The loss of anchor points with ablation of Tdn and Tdn/Jct coincided with a loss of polymerized CASQ that ultimately determined the size and shape of the jSR cisternae. Importantly, the severity of the anchor's disruption was mirrored by its functional effects on intracellular Ca^2+^ homeostasis. Thus, despite the similarities between the two proteins, triadin and junctin in skeletal muscle are not structurally and functionally equivalent.

## Materials and Methods

### Ethics Statement

All experiments on animals from creation of null and double mice to establishment of their structural and physiological phenotypes were conducted using protocols approved by the institutional animal care and use committees at the Harvard Medical School.

### Generation of null mice

Triadin-null (Tdn-null) and Junctin-null (Jct-null) mice were generated as described previously [30], [37]. Double null Tdn/Jct mice were obtained by breeding of Tdn-null and Jct-null mice. Genotype was determined by polymerase chain reaction of tail DNA. As previously described for the single genotype mice the newly generated Tdn/Jct double-null mouse did not exhibit embryonic or birth lethality. Although compared to WT animals the skeletal muscle from double-null mouse seemed to present a slight reduction in overall mass this did not translate in an obvious gross functional phenotype.

### Membrane vesicle preparation and Immunoblotting

Crude membrane preparations from lower limb muscle and primary myotubes were prepared as described previously [30]. Proteins were separated in SDS-polyacrylamide gel electrophoresis [60] and transferred to PVDF membrane. Expression of specific proteins was tested by incubation of immunoblots with poly- or monoclonal antibodies against; RyR1 (34C, ISHB, University of Iowa), Calsequestrin, FKBP-12/12.6 and DHPR α_1S_ (MA3-913, PA1-901 and MA3-927, respectively, from Thermo Scientific, Rockford IL), Junctin (1E6, gift from Dr. L. Jones) Junctophilin-1 and HRC (HPA009413 and HPA004833, Sigma) and GAPDH (FL-335 from SCBT, Santa Cruz CA). Membranes were then incubated with either goat anti-mouse or goat anti-rabbit horseradish-peroxidase-conjugated, secondary antibody and developed with SuperSignal ultra chemiluminescent substrate (Pierce, Rockford IL) and the intensity of the signal collected using a Kodak Imaging Station 4000MM PRO (Carestream Health, Rochester, NY). Band identification and densitometry of the identified proteins were performed using Kodak MI Software (version 4.5.1 ES). Net band intensities of unsaturated blots were normalized to GAPDH expression (FL-335, SCBT, Santa Cruz CA) to correct for variations in protein loading between lanes and then expressed as fraction of the WT signal.

### Electron microscopy

C57Bl/6 WT, Jct-null, Tdn-null and Tdn/Jct-null mice at 3–4 months of age were sacrificed by cervical dislocation. EDL and soleus muscles were dissected, pinned to a Sylgard dish (Dow Corning) at resting length and fixed. Sternomastoid was fixed in situ before dissecting. The muscles were fixed with 3.5% glutaraldehyde in 0.1 M Sodium Cacodylate buffer (pH 7.2) at room temperature and stored in fixative at 4°C for variable periods of time. Muscle segments were post-fixed in 2% OsO_4_ in the same buffer for 1–2 hr at 4°C, en block-stained in saturated uranyl acetate and embedded in Epon 812. Sections (about 40 nm) were cut in Leica Ultracut R microtome (Leica Microsystem, Austria) using a Diatome diamond knife (Diatome Ltd. CH-2501 Biel, Switzerland) and stained with lead citrate solutions. Sections were imaged in FP 505 Morgagni Series 268D (Philips, Brno, Czech Republic) with Megaview III digital camera (Munster, Germany) and in Phillips 410 (Philips Electron Optics, Mahwak, NJ) with Hamamatsu C4742-95 digital camera (Advanced Microscopy Techniques, Chazy, NY) electron microscopes.

#### Preparation of Figures

Figures were mounted and labeled using Adobe Photoshop® v7.0.

#### Quantization

The area of the jSR cross sectioned profile was measure using NIH image (ImageJ 1.45) in randomly collected images taken at a magnification of 143,000. Dimensions of feet, anchors and SR-SR bridges were measured using Photoshop from images at a magnification of 143–184.000. Statistical differences were evaluated using a Student's t test for unpaired data (Excel Software (Microsoft) and Prism 4.0 (GraphPad)). Unless otherwise indicated, EM data are presented as mean ± standard deviation (SD).

### Cell culturing and Ca^2+^ imaging

Primary myoblasts were isolated from mouse skeletal muscles of each phenotype and differentiated as described previously [30]. Ca^2+^ imaging was performed 5 days after differentiation in myotubes loaded with either 5 µM Fura-4F AM (Molecular Probes, OR) in imaging buffer (125 mM NaCl, 5 mM KCl, 2 mM CaCl_2_, 1.2 mM MgSO_4_, 6 mM Glucose, and 25 mM Hepes/Tris, pH 7.4). Sensitivity to K^+^-depolarization and caffeine-activation were determined by 5 s perfusion with 5–6 volumes of KCl (15 mM to 60 mM) or caffeine (3 mM to 40 mM). Cell were alternately excited at 340 nm and 380 nm at a rate of 4 Hz with a DG4 multi-wavelength light source and the fluorescent emission at 510 nm captured from regions of interest within each myotube using a Stanford Photonics 12 bit digital intensified CCD. SR Ca^2+^ content of cultured myotubes was estimated from the peak amplitude of the Ca^2+^ release signal induced by 10 µM cyclopiazonic acid (CPA) from cells loaded with 5 µM Fura-2F AM. Data are displayed and analyzed using QED imaging software (QED Software, Pittsburgh PA). Fluorescence signals are expressed as ratio of signals collected at alternating 340 nm/380 nm excitation wavelengths.

### Excitation-coupled Ca^2+^-entry (ECCE)

Ca^2+^ entry during depolarization was estimated from the rate of dye quench by Mn^2+^ entry in myotubes loaded with 5 µM Fura-2-AM according to [61], [62]. To prevent Ca^2+^ release from SR stores during depolarization cells were incubated overnight with 12 µM ryanodine to block RyR1 activation. Cells were depolarized with 80 mM KCl in Ca^2+^-free imaging buffer containing 0.5 mM MnCl (40 mM NaCl, 80 mM KCl, 2.2 mM MgSO_4_, 6 mM Glucose, and 25 mM Hepes/Tris, pH 7.4) at the isosbestic wavelength for Fura-2 (360 nm) and fluorescence emission at 510 nm was then captured from regions of interest within each myotube at a rate of 15 frames per second (fps).

### Resting free Ca^2+^ measurements

Determination of myoplasmic resting free Ca^2+^ concentrations of myotubes was performed with double-barreled Ca^2+^-selective microelectrodes assembled with ETH129 resin as described previously [63].
